# Social inequalities and correlates of psychotropic drug use among young adults: a population-based questionnaire study

**DOI:** 10.1186/1475-9276-7-3

**Published:** 2008-01-19

**Authors:** Nearkasen Chau, Michèle Baumann, Bruno Falissard, Marie Choquet, the Lorhandicap group

**Affiliations:** 1 Inserm, U669, Paris, F-75014, France; 2 Univ Paris-Sud, U669, Paris, F-75014, France; 3 Univ Paris Descartes, Paris, F-75014, France; 4 University of Luxembourg, INtegrative research unit on Social and Individual DEvelopment (INSIDE), Walferdange, Luxembourg; 5 AP-HP, Villejuif, F-94804, France

## Abstract

**Background:**

Use of psychotropic drugs is widespread in Europe, and is markedly more common in France than elsewhere. Young adults often fare less well than adolescents on health indicators (injury, homicide, and substance use). This population-based study assessed disparities in psychotropic drug use among people aged 18–29 from different socio-occupational groups and determined whether they were mediated by educational level, health status, income, health-related behaviours, family support, personality traits, or disability.

**Methods:**

A total of 1,257 people aged 18–29, randomly selected in north-eastern France completed a post-mailed questionnaire covering sex, date of birth, height, weight, educational level, occupation, smoking habit, alcohol abuse, income, health-status, diseases, reported disabilities, self-reported personality traits, family support, and frequent psychotropic medication for tiredness, nervousness/anxiety or insomnia. The data were analyzed using the adjusted odds ratios (ORa) computed with logistic models.

**Results:**

Use of psychotropic drugs was common (33.2%). Compared with upper/intermediate professionals, markedly high odds ratios adjusted for sex were found for manual workers (2.57, 95% CI 1.02–6.44), employees (2.58, 1.11–5.98), farmers/craftsmen/tradesmen (4.97, 1.13–21.8), students (2.40, 1.06–5.40), and housewives (3.82, 1.39–10.5). Adjusting for all the confounders considered reduced the estimates to a pronounced degree for manual workers (adjusted OR 1.49, non-significant) but only slightly for the other socio-occupational groups. The odds ratio for unemployed people did not reach statistical significance. The significant confounders were: sex, not-good health status, musculoskeletal disorders and other diseases, being worried, nervous or sad, and lack of family support (adjusted odds ratios between 1.60 and 2.50).

**Conclusion:**

There were marked disparities among young adults from different socio-occupational groups. Sex, health status, musculoskeletal diseases, family support, and personality traits were related to use of psychotropic drugs. These factors mediated the higher risk strongly among manual workers and slightly among the other groups.

## Background

Psychotropic drugs are widely used in Europe and markedly more common in France than elsewhere [1,2]. They have been shown to influence many aspects of health (including the growth and development of children, general health status, cancer, and quality of life) and to have major socio-economic consequences [3-5]. Frequent psychotropic drug use has a strong long-term association with injury [6-9].

Young adulthood is an important period of development and adaptation to working life. It is the age at which health indicators such as injury, homicide, and substance use, reach a peak, with levels higher even than those among adolescents [10]. Young people in employment are also at markedly greater risk of occupational injury than other age-groups [6]. Longitudinal data indicate that health risk increases and access to health care decreases from the teen and adult years for most US race/ethnic groups, and that relative rankings on a diverse range of health indicators vary by sex and race/ethnicity (as do patterns of change) – leading to fluctuating patterns of disparity over time [11]. In the last decade, young adults in Switzerland have been reported to be at increased risk of work-related injury, overweight or obesity, inter-personal violence, and regular use of alcohol, cigarettes and cannabis [12]. Even so, the health issues of young adulthood have received relatively little attention compared with those of adolescence, despite similarities in critical issues [10].

It is important to investigate the use of psychotropic medication and its correlates among young adults. People who begin working at an early age tend to work in less good environments, be under greater pressure, and experience more cumulative job stress, leading to fatigue, work-related stress reactions, psychological and physical overload, job dissatisfaction, health problems, and physical and mental disabilities [13-17] and consequently to psychotropic drug use [1,18-20]. In the European Union, 3.6% of the total burden of disease is directly related to the work environment [21]. In France, one-third of the working population use medications or other legal psychoactive substances in order to cope with work-related difficulties [22]. Psychotropic medications are used by many socio-occupational groups, but their administration is particularly common among manual workers [22].

Some material and psychosocial factors (low educational level, low income, smoking, alcohol abuse, lack of family support, certain personality traits) have been shown to influence health status, injury, disability, and mortality among various populations [6,14,23,24]. It would be interesting to investigate whether those factors, altered heath status, diseases, and disabilities relate to psychotropic drug use in young adulthood. In the literature, characteristics such as age, sex, body weight, smoking, alcohol use, physical symptoms and diseases, psychological factors, behaviour, personality, family support, and quality of life have been correlated with psychotropic medication [1,13,25-29] but no study has focused on young adults. It should be noted that smoking and alcohol use contribute to 9.0% and 8.4%, respectively, of the total burden of diseases in the European Union [21].

A key question is whether there are disparities in psychotropic drug use among young adults from different socio-occupational groups and, if so, whether they are mediated by the factors listed above. Inequalities in health due to material and psychosocial factors are of current concern throughout Europe [30-33]. In Italy, their reduction is part of the 1999–2000 national plan. A strategy for health that incorporates objective measures of equality has been established in Sweden. The government of the United Kingdom has reduced inequalities in health among children, and between socio-economic groups in general [34]. In France, reduction of inequalities in health has been an objective of a law related to public health policy.

There is considerable debate over the models used to explain inequalities in health, particularly with regard to whether they are mediated by social determinants systematically related to socio-economic status [32,33], with particular emphasis on material deprivation and psychosocial mechanisms [31]. Information about inequalities and patterns of risk for psychotropic medication in this context may aid the design of preventive measures, and help practitioners and other relevant professionals to provide appropriate health care and to monitor the subjects most at risk. To our knowledge, no such investigations have been conducted in young adults.

The present study assessed frequent use of psychotropic drugs among young adults (aged 18–29 years) from various socio-economic groups in northeast France, and investigated whether discrepancies were mediated by low educational level, not-good heath status, musculoskeletal disorders and other diseases, low income, smoking, alcohol abuse, lack of family support, certain personality traits, or disability.

## Methods

The initial sample consisted of everyone aged 15 years or more living in 8,000 randomly selected households in the Lorraine region of north-eastern France (2.3 million inhabitants). Only households with a telephone were eligible.

Before the initial survey, a 3-month media campaign (television, print, and radio) was conducted in order to raise awareness. The investigation was approved by the *Commission Nationale d'Informatique et Libertés*, and written informed consent was obtained from respondents.

The study protocol included an application to participate, which ascertained the number of people in the household, followed by three standardized self-administered questionnaires mailed at 1-month intervals, each with a covering letter and a pre-paid reply envelope. When the number of eligible individuals in a household was unknown, two questionnaires were sent initially, followed by another one later. Adolescents were free to ask their parents about any questions they did not understand. Questions covered: sex, date of birth, height, weight, educational level, occupation (coded according to the classification of the Institut National de la Statistique et des Etudes Economiques, Paris), smoking habit, excess alcohol use, perceived income, perceived health-status, various diseases diagnosed by a physician, reported disabilities according to the WHO international classification [35], self-reported personality characteristics, family support, and psychotropic drug use.

Underweight and overweight were defined as body mass index lower than 18.5 kg/m^2 ^and higher than 25 kg/m^2 ^respectively [36].

Alcohol abuse was defined using the Deta questionnaire (at least two positive responses to four items: (i) consumption considered excessive by the subject; (ii) consumption considered excessive by people around the subject, (iii) subject wishes to reduce consumption, and (iv) consumption on waking) [6,14,37]. With regard to self-reported personality characteristics, subjects were asked whether they considered themselves: worried, sociable, calm, aggressive, solitary, organised, nervous, or sad (Yes/No) [26,38,39]. Self-perceived health-status was addressed in the question: 'According to you, your health status is ...' (Good/Average/Poor/Bad). For perceived income, subjects were asked whether they considered themselves: comfortable or well off; earning just enough; coping, but not easily; or getting into debt. Family support was addressed in the question: 'Are you satisfied or dissatisfied with support from your family over the last two months?' (Very satisfied/Rather satisfied/No opinion/Rather dissatisfied/Very dissatisfied). Lack of family support was defined as 'Very dissatisfied' or 'Rather dissatisfied'.

The following categories of disabilities were considered: (a) sensory disabilities, with two items – vision and hearing; and (b) cognitive disabilities, with four items – concentration and attention; orientation; problem solving; and memory. Subjects were asked to 'indicate the response which corresponds to your ability in the following activities during the last 8 days...' (Without difficulty/With some difficulty/With several difficulties/Unable to comment).

With regard to psychotropic drugs, subjects were asked whether they usually frequently used medication (prescribed and/or non-prescribed) for tiredness, nervousness/anxiety, or insomnia (Yes/No) [7,8,26,38].

Nine occupational categories were considered: (1) upper occupations (intellectual professionals, senior managerial staff and administrators, medical doctors, independent professionals, engineers); (2) intermediate occupations (managerial staff, school teachers, skilled technicians, medical workers and social workers); (3) manual occupations (skilled manual workers, farm workers, semi-skilled manual workers, unskilled manual workers); (4) employees; (5) farmers (farm managers)/craftsmen/tradesmen (independent shop or business owners); (6) other employed people and those whose occupation is unknown; (7) students; (8) housewives; and (9) unemployed people [6,7,40]. Educational level was categorized into 'Primary school only', 'Middle and high school' and 'University'.

Of the 8,000 households included in the sample, mailings to 193 (2%) were lost (due to addressing error or death). Of 7,807 households contacted, 3,460 (44.3%) participated (all eligible members of the family took part in 86% of those). In total, 6,234 subjects filled in a questionnaire; 18 were of unknown sex or age, leaving 6,216 subjects who were similar in age and sex distribution to the population of Lorraine (Table 1). The subpopulation of interest here comprised 1,257 young adults aged 18–29.

**Table 1:** Distribution according to sex and age of the sample studied and of the Lorraine general population [54] (%)

	The sample studied	The Lorraine general population
No. of subjects	6,216	1,848,579
Percentage of women	52.4	51.5
Age (yr)		
15–19	5.4	9.6
20–24	8.0	9.8
25–29	9.7	9.7
30–34	10.4	9.6
35–39	10.5	9.6
40–44	7.9	9.3
45–49	8.5	5.9
50–54	6.0	6.6
55–59	6.3	6.8
60–64	7.2	6.6
65–69	7.5	5.7
70 or over	12.6	10.8

Only people aged 15 or more were considered.

### Statistical analyses

The outcome variable was psychotropic drug use (all types combined). Independent variables were: socio-occupational category, educational level, underweight, overweight, being a current smoker, alcohol abuse, not-good health status, musculoskeletal disorders and other diseases, hearing disability, visual disability, cognitive disabilities, being worried, not sociable, not calm, aggressive, solitary, not organised, nervous, or sad, living situation, lack of satisfaction with family support, and low perceived income.

First, crude odds ratios (OR) and 95% confidence intervals (CI) were used to assess the relationships between various factors and psychotropic drug use. Then two rounds of logistic regression analyses were carried out with two sets of independent variables and covariates: the first round included socio-occupational category and sex only, and the second round included all the risk factors studied which were related to psychotropic drug use with *p *< 0.10. In these analyses the upper and intermediate occupations were combined and used as the reference group (both contained a small number of subjects and the risk for psychotropic drug use was the same) (Table 2).

**Table 2:** Relationships between various risk factors and frequent psychotropic drug use in 1,257 young adults aged 18–29: crude relative risk and 95% CI

	No. of subjects	%	Crude odds ratio	95% CI
Women (vs. men)	712	56.6	2.08***	1.44–3.01
*Socio-occupational category: vs. managers, intellectual professionals *(73)				
Intermediate professionals	58	4.6	0.94	0.20–4.38
Manual workers	150	11.9	2.20	0.71–6.81
Employees	262	20.8	2.93*	1.01–8.50
Farmers, craftsmen and tradesmen	17	1.4	3.70	0.74–18.4
Other occupations or unknown	92	7.3	1.87	0.55–6.34
Students	463	36.8	2.52§	0.89–7.16
Housewives	53	4.2	5.05**	1.53–16.7
Unemployed people	89	7.1	1.94	0.57–6.58
*Educational level: vs. university *(413)				
Middle and high school	750	60.0	0.93	0.65–1.35
Primary school	94	7.5	1.11	0.58–2.14
Underweight vs. 'normal' (873)	95	9.8	1.31	0.73–2.36
Overweight vs. 'normal' (873)	289	24.9	0.81	0.53–1.25
Current smoking	476	37.9	1.52*	1.08–2.13
Alcohol abuse	97	7.7	1.46	0.83–2.57
Not-good health status	264	21.0	3.54***	2.49–5.05
*Diagnosed diseases vs. disease- free *(724)				
Musculoskeletal disorders (MSD)	193	15.3	1.96**	1.22–3.15
Other diseases	198	15.8	1.82**	1.13–2.94
Both MSD and other diseases	142	11.3	3.90***	2.47–6.17
Hearing disability	61	4.8	1.40	0.82–3.18
Visual disability	128	10.2	2.11**	1.33–3.35
Cognitive disability (at least one)	336	26.7	2.04***	1.44–2.89
*Self-reported personality traits*				
Worried	347	27.6	2.90***	2.06–4.09
Not sociable	557	44.3	1.34§	0.95–1.87
Not calm	880	70.0	1.99***	1.30–3.03
Aggressive	116	9.2	1.68*	1.01–2.78
Solitary	165	13.0	0.69	0.39–1.20
Not organised	861	68.5	1.02	0.71–1.46
Nervous	441	35.1	3.11***	2.20–4.39
Sad	56	4.5	5.24***	2.98–9.23
*Living situation: vs. living with family or in a couple *(1012)				
With friends	20	1.7	0.87	0.20–3.78
Alone	147	12.5	1.60§	1.00–2.56
Lack of family support	116	9.2	2.29***	1.42–3.67
Low perceived income	603	48.0	1.20	0.86–1.68

*p < 0.05, **p < 0.01, ***p < 0.001, § NS but *p *< 0.10.

Underweight: body mass index < 18.5 kg/m^2^, overweight: body mass index > 25 kg/m^2^, 'normal' weight: 18.5 kg/m^2 ^≤ body mass index < 25 kg/m^2^.

In parentheses: no. of subjects.

## Results

Frequent use of psychotropic drugs was common (12.2% overall, 7.6% for nervousness or anxiety, 4.6% for fatigue, and 2.6% for insomnia) and twice as high among women than men (odds ratio adjusted for occupation 2.03, 95% CI 1.38–3.01).

The characteristics of the subjects are shown in Table 2. Factors significantly related to psychotropic drug use were: sex, socio-occupational category, current smoking, not-good health status, musculoskeletal disorders and other diseases, visual disability, cognitive disabilities, being worried, not calm, aggressive, nervous, or sad, and lack of satisfaction with family support.

The findings of the two rounds of analyses are presented in Table 3. Compared with upper/intermediate occupations, markedly high and significant odds ratios adjusted for sex were found for manual workers (2.57), employees (2.58), farmers/craftsmen/tradesmen (4.97), students (2.40), and for housewives (3.82). Adjusting for all the confounders considered reduced the estimates slightly, and the odds ratios remained significant or close to significant, with the exception of manual workers (1.49, non-significant). Significant confounders were: sex, not-good health status, musculoskeletal disorders and other diseases, being worried, nervous or sad, and lack of family support.

**Table 3:** Relationships between various risk factors and frequent psychotropic drug use in 1,257 young adults aged 18–29: adjusted odds ratio and 95% CI computed with logistic model

Logistic regression model including socio-occupational category and sex only		
Socio-occupational category: vs. managers, intellectual and intermediate professionals (131)		
Manual workers	2.57*	1.02–6.44
Employees	2.58*	1.11–5.98
Farmers, craftsmen and tradesmen	4.97*	1.13–21.8
Other occupations or unknown	1.69	0.60–4.74
Students	2.40*	1.06–5.40
Housewives	3.82**	1.39–10.5
Unemployed people	1.74	0.62–4.89
Woman (vs. men)	2.03***	1.38–3.01
*Logistic regression model including all factors considered (Table 2) Socio-occupational category: vs. managers, intellectual and intermediate professionals *(131)		
Manual workers	1.49	0.56–4.03
Employees	2.22§	0.92–5.36
Farmers, craftsmen and tradesmen	5.40*	1.14–25.4
Other occupations or unknown	1.49	0.51–4.39
Students	2.22§	0.95–5.18
Housewives	3.23*	1.09–9.60
Unemployed people	1.50	0.51–4.44
Woman (vs. men)	1.60*	1.05–2.45
Current smokers	1.38§	0.94–2.01
Not-good health status	2.01***	1.34–3.02
*Diagnosed diseases vs. disease free*		
Musculoskeletal disorders (MSD)	1.80*	1.08–3.02
Other diseases	1.32	0.78–2.23
Both MSD and other diseases	2.13**	1.25–3.62
Visual disability	1.44	0.85–2.46
Cognitive disability (at least one)	0.84	0.55–1.29
*Self-reported personality traits*		
Worried	1.83**	0.23–2.73
Not sociable	1.27	0.87–1.86
Not calm	1.15	0.71–1.85
Aggressive	1.06	0.60–1.89
Nervous	1.95***	1.29–2.95
Sad	2.50**	1.30–4.82
Living alone	1.65§	0.97–2.80
Lack of family support	2.03**	1.19–3.45

*p < 0.05, **p < 0.01, ***p < 0.001, § NS but *p *< 0.10.

^a ^Also adjusted for socio-occupational category.

The logistic model considered only those factors related to psychotropic drug use with p < 0.10 (Table 2).

## Discussion

The present study demonstrates marked differences between socio-occupational groups in frequent use of psychotropic drugs (for fatigue, nervousness, anxiety, or insomnia) among young adults aged 18–29. Use was strongly mediated among manual workers, and slightly mediated among the other socio-occupational groups, by not-good health status, musculoskeletal disorders/other diseases, lack of family support, and certain personality traits such as being worried, nervous or sad. Current smoking, and visual and cognitive disabilities had significant crude odds ratios but they became non-significant when controlling for all factors considered. This could be explained by the interdependence of factors.

This study reports that frequent psychotropic drug use was common among young adults. Comparison with other investigations is difficult because of variations in the populations studied, the psychotropic drugs considered, and the methodological approaches used. In addition, few researchers have focused on young adults. In France, 9.3%, 16.1% and 5.9% of adults (aged 18–75) used antidepressants, sleeping pills, and drugs to improve their physical/intellectual performance [15]. One-third of French workers used drugs for work-related reasons, 20% to feel better, 12% to control an awkward symptom, and 18% to relax after a difficult day's work [22]. The ESEMeD study focused on the use of antidepressant, anxiolytic, and antipsychotic or mood-stabilizing drugs in adults (aged 18+) [1]. It reported a prevalence of 19.2% in France, 15.5% in Spain, 13.7% in Italy, 13.2% in Belgium, 7.4% in the Netherlands, and 5.9% in Germany. Bruffaerts et al. [41] reported that about 19% of Belgian people aged over 18 use a psychotropic drug.

Our results confirm the well-known sex ratio [1,26,28]. Women and female adolescents are more likely than their male counterparts to have any mental disorder and to take psychotropic drugs [1,2,26], to be given such drugs following a medical consultation, to receive longer courses, and to renew the treatment [42]. It should be noted that we found a crude odds ratio of 2.08 and an adjusted odds ratio of 1.60 when controlling for socio-occupational category and other confounders, whereas the ESEMeD study (conducted in six European countries) reported an adjusted odds ratio of 2.1 (95% CI 1.9–2.4) when controlling for age, marital status, educational level, employment, disability, illness leave, urban/rural location, and country [1]. This suggests that the sex ratio was only partly explained by these risk factors or confounders. Sex difference may in part be attributable to a higher incidence of depressive symptoms among females, and their greater willingness to seek medical help [2].

The present study reveals that housewives and students are at high risk for psychotropic drug use, whereas unemployed young adults are not. Overall, housewives had less good health status than female workers did, although this pattern was more consistent for women of low educational level [43]. University students report higher levels of anxiety and depressive symptoms than are seen in general population norms [44]. Several stressors, such as financial problems, and academic pressures and their consequences on social life, have an adverse effect on the mental health of students [45].

An important finding of our study is that there are marked disparities in psychotropic drug use among young adults from different socio-occupational groups. Manual workers, employees, farmers, craftsmen, and tradesmen were at high risk compared with upper/intermediate professionals. This was expected as work conditions, diseases, occupational injuries, impairments, disabilities, and health-related behaviours also differ greatly [6,15,18,23,37,46] and may increase psychotropic drug use [1,15,37]. Similar differences were observed between socio-occupational groups in impairments among subjects aged less than 40 years [14]. Health hazards at work are still a major determinant of poor health [30]. In the European Union, 3.6% of the total burden of disease is directly related to work environments [30]. In France, the disparities between social groups in morbidity, mortality and premature mortality are higher than in the other western European countries [47]. Poor working conditions, and particularly cumulative job stress, lead to physical and mental disturbances, and consequently to psychotropic drug use [18]. As noted above, one in every three French workers uses medications or other legal psychoactive substances in order to cope with work-related difficulties, and such use is more common in employees and manual workers [22]. Fatigue is related to the physical demands of job[13]. The volume of services provided and job dissatisfaction are associated with hypnotic and tranquillizer use [19]. Work-related fatigue is generated by repeated episodes of adverse work experience and leads to the development of stress reactions, psychological overload, and health problems [16]. Unemployed people here had a crude odds ratio of 1.94, but it was not significant. However, that may relate to a lack of statistical power, suggesting that a study with a larger sample is needed.

As reported by other authors [10,11], we found that not-good heath status and diseases were common in young adults, and that they were strongly associated with psychotropic drug intake. Musculoskeletal disorders were most common and had a marked effect on psychotropic medication. The high risk among subjects with not-good health status or musculoskeletal disorders is of paramount importance as their prevalence is high in the population of all ages [6,46]. Our results are consistent with those of other studies that have shown that psychotropic medication is associated with sick leave due to illness, severe physical symptoms, and disability [1,48]. Not-good health status, disease, and psychological distress are strongly related to onset of fatigue and sleep problems [25,49]. The presence of disease is, along with sensory or cognitive disabilities, a long-term risk factor for injuries and falls [6,23].

Our investigation shows that self-reported personality traits were related to psychotropic drug use, and that the simple items used were pertinent. Subjects who described themselves as nervous, not calm, worried, aggressive or sad were at increased risk. It may be that these self-reported personality traits may be interpreted as a sort of a justification for the use/abuse of psychotropic medications This finding was expected because self-reported personality traits are associated with smoking, alcohol consumption, illicit drug use, and occupational injury [38,39].

We found that lack of family support was strongly associated with psychotropic medication among young adults. The role of the family in substance use is well documented, but not among this age group [29]. We also demonstrated that visual and cognitive disabilities were associated with psychotropic drug use in univariate analysis but not in multivariate analysis taking into account sex, socio-occupational category, health status, disease, family support, and personality traits. These results suggest that the increased risks associated with visual and cognitive disabilities could be attributed to those confounders, indicating that preventive measures to reduce psychotropic medication use among young adults may need to be focused on health status, diseases, family support, and personality traits rather than low education level, disability, and low income which are mainly related to socio-economic groups. The ESEMeD study found that psychotropic drug use was related to lower levels of education, but health status, disease, family support, and socio-occupational category were not considered [1]. A low education level is well known to be associated with disability [23], membership of a lower social class, and with adverse work conditions, lifestyle and living conditions.

Our study failed to detect an association between frequent psychotropic drug use and overweight, underweight, or alcohol abuse. McElroy *et al*. [50] stated that obesity is associated with depressive disorders, but most overweight and obese people in the community do not have mood disorders. Bültmann *et al*. [25] also found an increased risk of fatigue in underweight women. Eating alone, social isolation, and stressors are the main reasons for low weight reported by the subjects concerned, mainly due to poor nutritional status [51]. The present study found a significant relationship between smoking and psychotropic drug use in univariate analysis and a relationship close to significance in multivariate analysis taking into account all covariables. Smoking is associated with low socio-economic group, low educational level, low income, living alone, occupational and domestic injuries, and premature death (≤ 70 yr) [8,40,52]. Preventive measures to reduce psychotropic medication may consequently need to be focused on smokers. Alcohol abuse affected 7.7% of young adults; it had a crude odds ratio of 1.46 (slightly lower than that for smoking (1.52)) that was non-significant, again perhaps because of a lack of statistical power, suggesting that a study with a larger sample is needed.

Our study demonstrates that disparities in frequent use of psychotropic drugs were mediated by not-good health status, musculoskeletal disorders, lack of family support, and certain personality traits, all of which are generally chronic or long-lasting conditions, particularly among lower socio-economic groups. Therefore, psychotropic drug use generated by them would also last for a long time. Consequently, general practitioners may need to monitor those of their patients most at risk. According to the ESEMeD study, a significant proportion of individuals, both with and without mental disorders, are inappropriately treated [1]. These results confirm the roles of material conditions and psychological factors in social inequalities in health [15,17,30,31,37,53] but they also point out a relatively important role of personality in psychotropic medication. These findings may help us understand socio-occupational inequalities in health, which, it should be noted, are currently an area of considerable interest and a preoccupation among policy officials in most European countries and elsewhere [17,30,31].

Any selection bias here would be small: 96% of households had telephones at the time of the study, and only 16% had confidential addresses. Discussions before the survey, for example with associations of people with disability, suggested that neither is likely to be related to health status or living conditions. The age and sex distributions of the sample reflect those of the general population of Lorraine [54]. The percentage of manual workers (18%) was similar to that of the Lorraine population (21%) [54]. Note also that the incidence rate of occupational injury and the prevalence of various types of diseases and disabilities, for example, are similar to those of the general population [6,8,14,55]. The quality of the completed questionnaires was very good. All the factors studied had been validated and used in other investigations [6,15,26,37-39].

The present study had some limitations. First, the psychotropic drugs considered included those for fatigue, nervousness, anxiety, and/or insomnia. Fatigue is associated with psychological distress and absence due to sickness [16,56], and sleep problems are related to depression, pain, and hypnotic-sedative use [48]. Second, the participation rate was modest although it was similar to that achieved in similar surveys in France [1,57]. Third, as the study used a self-administered questionnaire, the results should be interpreted with caution, particularly given a possible selection bias. However, the self-administered occupational health history questionnaire is reliable and valid [58]. The non-response bias in mailed health surveys is small [59]. In population-based studies, self-assessment of vision is similar between participants and non-participants [60], and self-assessment of memory is generally valid [61].

## Conclusion

The frequent use of psychotropic drugs was common and there were marked disparities between young adults from different socio-occupational groups. It was related to sex, health status, musculoskeletal disorders, lack of family support, and certain personality traits. These factors strongly mediated the higher risk among manual workers and slightly mediated it among the other socio-occupational groups. Preventive measures to reduce psychotropic medication and inequalities in health should include interventions to improve work conditions, health status, and family environment, and to prevent musculoskeletal disorders. General practitioners may help young adults concerned to be more aware of the risks, to seek appropriate health care, and to take remedial measures.

## Competing interests

The author(s) declare that they have no competing interests.

## Authors' contributions

NC participated in conceiving and carrying out the study, and was the investigator with most responsibility for writing the manuscript.

MB participated in conceiving the study and writing the manuscript.

BF participated in conceiving the study and writing the manuscript.

MC participated in conceiving the study and writing the manuscript.

The Lorhandicap group, which carried out the study of which this work is a part, followed and reviewed the manuscript.

All authors read and approved the final manuscript.
